# Oral Manifestations of Inflammatory Bowel Disease and the Role of Non-Invasive Surrogate Markers of Disease Activity

**DOI:** 10.3390/medicines7060033

**Published:** 2020-06-16

**Authors:** Davide Giuseppe Ribaldone, Selvaggia Brigo, Michela Mangia, Giorgio Maria Saracco, Marco Astegiano, Rinaldo Pellicano

**Affiliations:** 1 Department of Medical Sciences, University of Turin, 10126 Turin, Italy; mickela.89.mm@gmail.com (M.M.); giorgiomaria.saracco@unito.it (G.M.S.); 2 Bow Lane Dental Group, St George’s Hospital, Bupa Dental Care, London SW17 0QT, UK; selvy.brigo@gmail.com; 3 Unit of Gastroenterology, Molinette Hospital, 10126 Turin, Italy; marcoastegiano58@gmail.com (M.A.); rinaldo_pellican@hotmail.com (R.P.)

**Keywords:** inflammatory bowel disease, Crohn’s disease, ulcerative colitis, extra-intestinal manifestations, orofacial granulomatosis, tag-like lesions, cobblestoning, mucogingivitis, lip swelling, aphthous stomatitis

## Abstract

Inflammatory bowel disease (IBD), which includes Crohn’s disease (CD) and ulcerative colitis (UC), can be associated with several extra-intestinal manifestations requiring a multidisciplinary management both in terms of work-up and therapy. Oral lesions are common in patients with IBD, with a prevalence ranging from 5% to 50%. These can represent an oral location of IBD as well as a side-effect of drugs used to treat the intestinal disease. Oral manifestations, occurring in patients with IBD, can be divided in nonmalignant, specific, and non-specific ones, and malignant lesions. While there is undoubtedly a need to search for an IBD in patients with oral lesions associated with intestinal symptoms, the work-up of those with an exclusive oral lesion should be personalized. Fecal calprotectin is a non-invasive marker of intestinal inflammation and may be used to select which patients need to undergo endoscopic examination, thereby avoiding unnecessary investigations. The pharmacological armamentarium to treat oral lesions associated with IBD includes topical or systemic corticosteroids, immunosuppressive agents, and biologic drugs.

## 1. Introduction

Inflammatory bowel disease (IBD) comprises chronic heterogeneous disorders of unknown etiology, resulting from multifactorial environmental precipitants in genetically susceptible individuals [1]. IBD are distinguished in two main phenotypes, Crohn’s disease (CD) and ulcerative colitis (UC), characterized by inflammation of the intestinal mucosa [2]. While UC affects the rectum and a variable extent of the colon, CD can involve any location of the gastrointestinal (GI) tract, from the oral cavity to the anus. Furthermore, up to 36% of patients with IBD may have extra-intestinal manifestations (EIM) [3,4,5] which can affect almost any organ of the body (eyes, joints, liver, pancreas, skin, blood, and mouth) [6,7]. 

The pathogenesis of IBD and EIM remain to be elucidated. Common genetic background (IBD1 gene [8], MHC allele HLADRB1*0103 [9], ABCB1 gene [10]) between the two conditions may be the predisposing cause, but the detailed mechanisms remain unknown, especially because the environmental triggers responsible for disease onset have not been elucidated. In fact, although many factors (such as infectious agents, diet, drugs, smoking) have been investigated, a precise causal agent, or a cluster of agents, has not been identified. In the last few years, a role of GI microbiota in the pathogenesis of IBD has been proposed [11]. The human GI microbiota consists of a wide variety of bacteria, viruses, fungi and other single-celled organisms. Although bacteria belong to four phyla (Firmicutes, Bacteroidetes, Proteobacteria, and Actinobacteria) the majority are from either Firmicutes or Bacteroidetes [12]. It remains to be clarified, in IBD patients, if there is a decrease in microbial diversity as a consequence of intestinal changes, or if this could play a role in the pathogenesis of these diseases. Because of the potential wide spectrum of involvement and of the genetic commonalities, it has been hypothesized that IBD represents a systemic disease with a predominantly intestinal manifestation [13].

## 2. Oral Lesions in Patients with IBD

The first cases of oral IBD manifestations were described in 1969 in two patients with CD [14]. In the last 50 years, the prevalence of oral lesions in patients with IBD has been reported to range from 5% to 50%, according to heterogeneous studies [15,16]. This wide range could be due to various reasons: first, the heterogeneity of the studies, including patients of different ages, ethnicities, and genetic backgrounds; second, the authors’ level of experience; third, the variability in the definition of lesions. The latter, in fact, could be a specific IBD lesion, but could also have iatrogenic origin, due to the drugs used to treat IBD. 

The prevalence of oral manifestations is higher in males and in children [16]. The higher incidence of upper GI tract involvement in children with CD, compared to adults, could explain the higher prevalence of oral lesions in pediatric CD [17]. Although oral lesions are generally more prevalent in CD (20%–50%) [18] than in UC (8%) patients, in some studies a significant difference was not observed [19]. Moreover, in adult CD patients, the prevalence rate of oral manifestations is higher in those with upper GI tract involvement and perianal disease [20].

Oral manifestations can occur either concomitantly with intestinal symptoms or before the presentation of IBD. In 60% of these patients, oral lesions may be the primary presenting sign, preceding GI manifestations [21,22]. Although the oral mucosa lesions and oral symptoms can be more severe during disease activity period, the correlation is not universal, and up to 30% of affected patients continue to suffer active oral manifestations (especially in the pediatric age group) despite remission of IBD [23]. Oral IBD manifestations can be divided into specific and not-specific lesions (Table 1), according to the presence of granulomas noted on the histopathology examinations [19,24]. It should be highlighted that some of these lesions can be considered an oral location of IBD while others are the result of nutritional deficiencies secondary to intestinal malabsorption.

For IBD, and for its oral manifestations, the pathogenesis remains unclear. Parallel to what has been reported on the potential role of microbiota in the pathogenesis of IBD and its oral location, it has been proposed that dysbiosis (term that means imbalance within the bacterial community) of salivary microbiota (with relative abundance of Streptococcus, Prevotella, Haemophilus, and Veillonella) may play a crucial role [25]. 

## 3. Specific Oral Lesions

Specific oral lesions are less common than non-specific ones. The main feature of specific lesions is the presence of non-caseous granulomas, observed only in patients with CD (CD with concomitant orofacial granulomatosis) [26]. Granulomas consist of a core of activated macrophages, some of which merge to form giant cells, surrounded by lymphocytes and fibrotic tissue. Granulomas are found in only 24%–61% of patients with CD, whereas in those with orofacial granulomatosis, granuloma formation in the oral lesions occurs in 70%–100% of cases, irrespective of coexisting CD. It has been reported that CD with concomitant orofacial granulomatosis is frequently associated with perianal lesions; furthermore, children with CD and concomitant orofacial granulomatosis show a more extensive disease phenotype and proximal GI tract involvement than CD patients with intestinal inflammation only [27].

The differential diagnosis should consider that granulomatous oral lesions can also occur in other diseases, including orofacial granulomatosis, sarcoidosis, mycobacterial infection, and foreign-body reactions.

The specific oral lesions include indurated tag-like lesions, cobblestoning, mucogingivitis, lip swelling with vertical fissures, and deep linear ulcerations (Table 1).

### 3.1. Clinical Characteristics

In cobblestoning, fissured swollen buccal mucosa with corrugation and hyperplastic appearance of the mucosa resemble a “cobblestone”. These lesions are usually detected in the posterior buccal mucosa and consist of mucosal-colored papules that produce firm plaques on the buccal mucosa and palate. In addition, indurated polypoid fringe-like lesions can be observed in the vestibule and in the retromolar region. Such lesions may cause pain and make speaking and eating difficult [28].

Mucosal tags and deep linear ulcerations (lip and tongue fissures) have hyperplastic edges, which can be firm or boggy to palpation. Attached gingiva and alveolar mucosa can become granulated, swollen, and hyperplastic with or without ulcerations. These lesions are mostly present in the labial and buccal vestibules and in retromolar regions.

In the setting of specific lesions, edema of the face, of one or both lips, and of the buccal mucosa has been described. The lips are the most commonly affected, and they are usually painless, tender, and firm to palpation [29]. Painful vertical fissures can occur in numerous patients with swollen lips; many microorganisms can be isolated in lip fissures [30]. 

Mucogingivitis can be present. The gingiva appears edematous, granular, and hyperplastic in CD, with or without ulcerations. The whole gingiva up to the mucogingival line might be involved [31].

A rare manifestation of CD could be represented by autoimmune changes of the minor salivary glands and dry mouth [32]. Chronic inflammatory processes near the parotid duct result in partial to total duct obstruction and cause dilated ducts and cyst formation, which can lead to the formation of cutaneous fistulas. All these changes can also lead to a reduction in saliva production and dry mouth [33].

Other specific manifestations include granulomatous cheilitis, macrocheilia, and palatal ulcer. In case of granulomatous cheilitis the main change is a chronic granulomatous inflammation, with edema and lumpy swelling of the lips. 

### 3.2. Diagnosis

The differential diagnosis of specific oral lesions includes syndromes presenting with multiple mucosal swellings, such as multiple hamartoma syndrome (Cowden disease), multiple endocrine neoplasia (MEN) 2B/III, neurofibromatosis, and idiopathic orofacial granulomatosis.

In the case of histologically confirmed oral granulomatous, the differential diagnosis includes foreign body reaction, allergic reaction to benzoate or cinnamon, and idiopathic orofacial granulomatosis. Systemic conditions associated with granulomatous inflammation include deep fungal infections, mycobacterial infections, sarcoidosis, and tertiary syphilis.

## 4. Non-Specific Oral Lesions

Non-specific oral lesions (Table 2) occur more frequently than specific lesions, so differential diagnosis can be difficult. These lesions may occur as result of chronic inflammation, malnutrition and malabsorption syndrome, or as a side effect of pharmacological treatment.

Patients with IBD and EIM may suffer from recurrent aphthous stomatitis more often than others; these lesions may occur in up to 10% of patients with UC and up to 20%–30% of patients with CD [34]. Apthae are shallow, round ulcerations with central fibrinous exudate surrounded by an erythematous border (“halo”). Aphthous stomatitis are not specific for IBD and may be observed in several other disorders including celiac sprue, human immunodeficiency virus (HIV)/acquired immune deficiency syndrome, autoimmune rheumatic disease (lupus, Bechet’s disease and Reiter’s syndrome), infections (herpes virus, cytomegalovirus), autoimmune bullous diseases, and common aphthae seen in the normal population. 

Angular cheilitis is characterized by erythema at the corners of the mouth with or without painful fissures and sores. It can be a consequence of anemia or fungal and bacterial infections [35].

Pyostomatitis vegetans (PV) is a rare, benign, chronic, mucocutaneous ulcerative disorder, considered the oral equivalent of pyodermatitis vegetans of the skin [36]. There is a frequent association between PV and IBD; it occurs in patients with UC more commonly than in those with CD and, in the former, is considered a specific marker of disease activity [37]. PV is characterized by erythematous and thickened oral mucosa with multiple pustules and superficial erosions. Multiple white or yellow pustules may rupture, and form folded, fissured appearances resembling a “snail-track”. The most affected areas are the labial gingiva, buccal and labial mucosa, and soft and hard palate. PV may present with oral ulcers (with possibly oral malodor). The differential diagnosis includes autoimmune pemphigoid diseases and infections [38]. The diagnosis of PV is based on the result of biopsy specimen obtained from the affected area. Microscopic sections show intraepithelial clefting and acantholysis. Within the spinous layer, the accumulation of eosinophils (intraepithelial abscesses) are also seen. The underlying connective tissue demonstrates the infiltration of mixed inflammatory cells [39]. Some authors have suggested that PV belongs to the spectrum of neutrophilic dermatoses or even represents an oral form of pyoderma gangrenosum [40].

In patients with IBD, caries and periodontal disease occur with a higher prevalence than in those without IBD. This is supported by the results of a meta-analysis, reporting that the risk of periodontitis is significantly increased in IBD compared to the control group and that it is more pronounced in UC than in CD [41]. In addition, the severity and extent of periodontitis is greater in IBD patients when compared to healthy controls [42], probably in association with the high expression of interleukin (IL)-18 in the serum of IBD patients with periodontitis [43]. The pathogenesis of periodontal disease, similar to that of IBD, involves local pathogens and the host immune-inflammatory response, and is influenced by genetic and environmental factors [44]. The increase in dental caries risk is thought to be associated to dietary habits, changes in saliva and microbiological conditions of the oral cavity, and deficient intestinal absorption of food substances. The malabsorption of vitamin D, which is common in IBD patients [45], may possibly be related to the complex multifactorial etiopathology of dental caries [46].

Other non-specific oral manifestations of IBD include stomatitis, glossitis, odynophagia and dysphagia, perioral dermatitis, diffuse pustules and non-specific gingivitis, lichenoid reactions, candidiasis, gingival hyperplasia, papillomatosis of the oral mucosa, pemphigus vegetans, persistent submandibular lymphadenopathy, recurrent buccal abscesses, and metallic dysgeusia [47].

## 5. Malignant Oral Manifestations

It is well-known that oral cancer is linked to several risk factors such as alcohol consumption, tobacco smoking, the male gender, and an age over 40 [48]. Furthermore, patients undergoing organ transplantation, especially in case of prolonged immunosuppression and those with HIV infection have an increased risk of developing oral cancer [49]. 

Oral cancer has a poor prognosis, with a 5-year survival <50%, higher for lip and lower for tongue and gingiva. Precancerous lesions are oral lichen planus, leukoplakia, erythroplakia, and erythroleukoplakia. In patients with IBD, oral cancerous and precancerous lesions have been reported. In particular, a higher risk of oral cavity tumors, especially of the tongue, has been reported in IBD patients compared to controls; furthermore, female have a higher risk than males. Based on the published literature, it is evident that patients with IBD are at high risk for developing these lesions, a phenomenon amplified by the increasing human papillomavirus (HPV) prevalence [50] reported in this population. The role of immunosuppression, considering that all the drugs used to treat IBD could be in theory involved in carcinogenic processes, remains to be defined. 

## 6. Diagnosis of IBD in Patients with Oral Manifestation

When an oral manifestation occurs, the presence of IBD should be suspected, even in absence of GI symptoms. Because oral lesions can precede the onset of IBD, a cooperation between specialists in oral medicine and gastroenterologists is required in order to allow an early diagnosis.

The diagnosis of IBD requires evaluation and a combination of clinical, laboratory, radiological, endoscopic, and pathological data [1]. Currently, an endoscopic examination with a colonoscopy and multiple biopsies is the gold standard to confirm IBD diagnosis. This procedure defines the extent and severity of mucosal involvement and allows biopsies collection for histological examination. Colonoscopy also enables the assessment of suspected stenosis in the distal ileum. However, it is considered an invasive and expensive procedure [51]. Imaging techniques for the diagnosis of suspected IBD include ultrasonography (US), computed tomography (CT) scanning, and magnetic resonance imaging (MRI) [52]. US is an accurate diagnostic tool to detect suspected CD and to evaluate disease activity (sensitivity 84%, specificity 92%); it is a widely available and non-invasive technique, but its accuracy is lower for the disease proximal to the terminal ileum [53]. MRI is a technique with a high accuracy for diagnosis and assessment of extension and activity of the disease (sensitivity 93%, specificity 90%); it is less dependent on the examiner’s experience and disease location compared to US [54]. CT and MRI have similar accuracy in evaluating the extent and activity of a disease. The three techniques detect fistulas, abscesses, and stenosis with high accuracy (sensitivity and specificity >0.80), although US may give false positive results for abscesses. US or MRI should be preferred over CT, particularly in young patients, due to its lack of radiation [53,54,55]. 

Blood tests include the “traditional” non-specific parameters of inflammation (i.e., erythrocyte sedimentation rate and C-reactive protein), and serologic markers of CD. Currently, among the latter, anti-neutrophil cytoplasmic antibodies (ANCA) and anti-Saccharomyces cerevisiae antibodies (ASCA) are the most used for diagnosis as well as for prognostic stratification of CD patients. ANCA are autoantibodies whose antigen targets are found mostly in azurophilic granules of neutrophils. In particular, in patients with inflammatory vasculitides, the major ANCA antigen targets are the proteinase-3, cytoplasmic granular with accentuation between nuclear lobes (cANCA), while in those with IBD, the target is the myeloperoxidase, which is a fine, homogenous, diffuse rim-like staining of perinuclear cytoplasm (pANCA) [56]. Positive p-ANCA are more common among UC patients, with a frequency of 40–80% in this cohort [57]. ASCA are antibodies directly against carbohydrate epitopes of phosphopeptidomannan, a 200 kDa glycoprotein of Saccharomyces cerevisiae cell wall, and have been specifically associated with CD, with 40%–60% sensitivity and 80% –90% specificity [58,59]. Recent studies reported the identification and preliminary evaluation of three new anti-glycan antibodies, called anti-laminaribioside carbohydrate antibodies (ALCA), anti-chitobioside carbohydrate antibodies (ACCA), and anti-mannobioside carbohydrate antibodies (AMCA). Some authors have assessed the accuracy of these tests showing that ASCA and ALCA antibody titers were significantly higher in CD patients than in controls. Moreover, significantly higher ACCA levels in patients with CD than in those affected by other GI diseases, were found; however, when comparing CD patients with healthy controls, a difference was not observed. Specificity and positive predictive value were always good candidates to confirm the association between considered markers (except ACCA) and CD [60,61]. The antimicrobial antibodies anti-I2 (CD related protein from *Pseudomonas fluorescens*), anti-Cbir 1(flagellin-like antigen), and anti-OmpC (*Escherichia coli* outer membrane porin C) seem to be associated with CD but the role and utility of these additional markers have yet to be determined [62].

The direct assay on feces for inflammatory markers has the potential to improve the accuracy of the serologic tests. Among candidate markers, fecal calprotectin has been investigated with promising results in terms of accuracy in distinguishing patients with an inflammatory disease compared to those without [63]. Calprotectin is a 36 kDa calcium- and zinc-binding protein found in blood cells and plasma; it accounts for about 60% of the cytosol in neutrophils [64], which are one of the major players in intestinal inflammation but also in monocytes and macrophages [65]. Calprotectin is not actively secreted from the neutrophils, but it is released following cell death or cell disruption [66]. The amount of fecal calprotectin is strongly correlated to the migration of neutrophils cells through the gut wall and its increase is proportional to the severity of inflammation. High levels of fecal calprotectin have been associated with IBD [67]. Moreover, calprotectin is fairly unaffected by therapy and enzymatic degradation and its level is stable outside the body at room temperature for up to 7 days, so it is a useful component for reliable enzyme-linked immunosorbent assay (ELISA) testing [68]. Based on a literature study, a fecal calprotectin of >200 µg/g permits the detection of CD in 50% of suspected cases. On the contrary, when it is <100 µg/g, further tests should be ruled out because the possibility of finding an inflammatory disease is low [69].

Fecal lactoferrin is an iron-binding glycoprotein of activated neutrophils. The fecal levels of lactoferrin are found to be strongly elevated in IBD patients as well as in those affected by infectious colitis. In patients with suspected CD, fecal lactoferrin has a sensitivity and specificity of 82% and 93%, respectively. However, the dosage of fecal lactoferrin seems to be less effective than that of calprotectin for the differential diagnosis between IBD and irritable bowel syndrome [70]. 

Granuloma on biopsy examination is the histological hallmark of both orofacial granulomatosis and oral CD. Orofacial granulomatosis is a complex condition characterized microscopically by a non-caseating granulomatous inflammation and macroscopically by chronic swelling of the lips, oral ulcers, and hyperplastic gingivitis, with no evidence of bowel and systemic involvement [71]. The differential diagnosis between these conditions, as well as from other disorders that can manifest with granulomas, requires clinical, invasive, and non-invasive approaches. Two key-points should be highlighted. First, it is known that the presence of oral granulomas could represent a manifestation of a silent intestinal CD. Previous studies have shown that half of the patients with these oral lesions had inflammatory involvement of mucosa, discovered on ileo-colonoscopy, in absence of specific GI symptoms. Histological abnormalities in intestinal mucosal samples are more likely to be found in case of an early onset of oral granulomatosis or in the presence of more severe oral inflammation [72]. Second, it is unknown if the presence of oral granulomas could be the early manifestation of a future CD of the GI tract [73]. In fact, the evolution of these lesions over time remains undetermined. Therefore, in absence of intestinal manifestations of CD, the diagnosis of isolated oral lesions may be difficult to ascribe to CD [74]. Some authors suggested that patients with orofacial granulomas should undergo a non-invasive test such as fecal calprotectin and eventually an endoscopic examination in the presence of GI symptoms for a differential diagnosis between an early oral manifestation of CD and orofacial granulomatosis [75].

As reported above, since there is no clear or expected pattern of intestinal IBD presenting with oral manifestations, the diagnostic algorithm should be personalized. 

Regarding the non-invasive approach, while it would seem easy to support an oral location of IBD in the presence of intestinal manifestations, in subjects without abdominal complaints, there are no data concordant with the possibility that non-specific parameters of inflammation can help to diagnose an oral IBD manifestation. A different consideration must be made for serologic markers of IBD and fecal calprotectin. Both, as above reported, are the expression of intestinal inflammation. Hence, these could be useful in contributing to the detection of silent forms of concurrent intestinal IBD in patients with suspected oral IBD. If intestinal IBD-like abnormalities are found, then this will support the diagnosis of concomitant oral location of IBD. Obviously, in the clinical setting, it is difficult (on both ethic and economic bases) to propose an endoscopic or an imaging approach in patients with exclusive oral granulomatosis, without a family history of intestinal disease. Only non-invasive biomarkers, due to their low costs and lack of radiation, are the most appropriate medical investigations to screen patients with suspected IBD.

## 7. Treatment of Oral Manifestations in Patients with IBD

The goals of treatment in IBD patients are to induce and maintain clinical and, when possible, endoscopic remission. Furthermore, the prevention of complications must be another main objective. 

**Figure 1:**
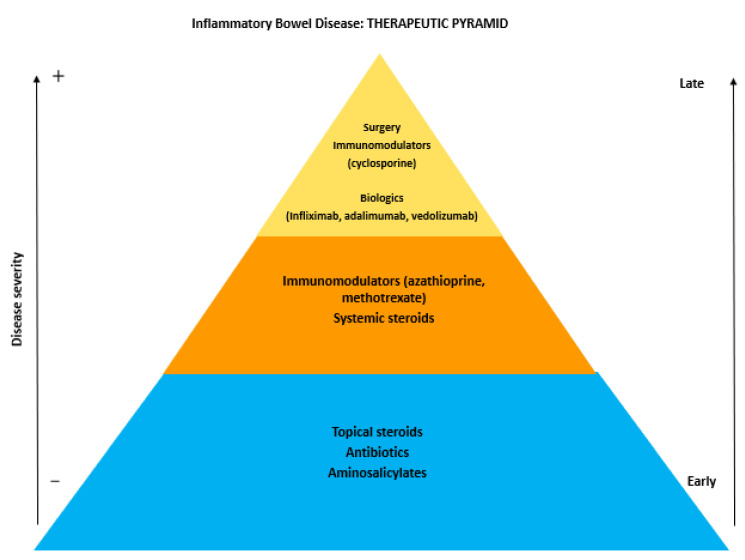
The therapeutic pyramid of inflammatory bowel disease.

The first-line therapeutic management of IBD should consider the severity, location, and behavior of the disease, as well as the presence of EIM. Subsequently, the assessment of response to previous treatments and the precedent side effects due to prescribed drugs should be considered. The therapeutic approach for IBD includes a standard treatment, with aminosalicylates, corticosteroids, immunosuppressive and immunomodulator agents (thiopurines, methotrexate and cyclosporin), and antibiotics. In the last few decades, the advent of biologic drugs, especially for patients resistant to or depending from standard treatment (in particular corticosteroids), has permitted a revolutionization of the natural history of IBD (Figure 1). 

The most diffuse approach in the clinical setting is based on a sequential strategy. This includes the initial use of aminosalicylates patients with mild diseases, and that of corticosteroids in patients with moderate to severe symptoms or in those who failed to respond to aminosalicylates. For maintenance therapy, after clinical remission has been obtained, immunomodulators (thiopurines) are used. Patients who failed steroid-induction or maintenance therapy with a thiopurine should be treated with a biologic drug.

Overall, in most patients, clinical remission of IBD is associated to healing of oral manifestations. When this does not occur, the treatment of oral manifestations mirrors the treatments utilized in the gastroenterological setting. The pharmacological armamentarium includes topical or systemic corticosteroids, immunosuppressive agents, and biologic (mainly anti-Tumor necrosis factor (TNF)-α) drugs. Topical treatment includes intralesional injections, mouthwashes, and ointments. This approach generally starts with corticosteroid ointments and/or mouthwashes and nonsteroideal anti-inflammatory pastes. In the case of non-response, an intralesional injection of corticosteroids is prescribed. If the patient’s symptoms do not improve, a systemic approach with corticosteroids is required [15]. In severe cases, for example of fistulising oral CD or difficult-to-treat aphthous stomatitis, treatment with biologic drugs has obtained satisfactory results [50]. 

## 8. Conclusions

In conclusion, while it is recommended to proceed with non-invasive investigations aiming to exclude IBD, in patients with oral granulomatosis and intestinal manifestations, the approach for subjects with only the former remains unclear and the diagnostic strategy should be personalized. Fecal calprotectin can be considered a noninvasive reliable marker of intestinal inflammation and may be used to improve the appropriate use of endoscopic examination in patients suffering from abdominal complaints, thereby avoiding unnecessary investigations. However, a diagnostic strategy with fecal calprotectin monitoring over time, in association to clinical follow-up, should be considered before endoscopy indication, highlighting that some organic intestinal diseases require an endoscopic approach and cannot be detected only by fecal calprotectin.

## Figures and Tables

**Table 1:** Specific oral lesions in patients with inflammatory bowel disease (IBD).

Lesion	Location	Features
Cobblestoning	Posterior buccal mucosa	Fissured swollen mucosa with corrugation and hyperplastic
Indurated tag-like lesions	Labial and buccal vestibules; retromolar region	Hyperplastic edge firm or boggy
Mucogingivitis	Whole gingiva	Gingiva edematous, granular and hyperplastic
Others: Lip swelling with vertical fissures Deep linear ulceration Edema of the face	Lips, tongue, buccal sulci, face	

**Table 2:** Non-specific oral lesions in patients with IBD.

Lesion	Location	Features
Aphtous stomatitis	Anywhere in the oral cavity	Shallow round ulcerations with central fibrinous exudate surrounded by an erythematous border
Pyostomatitis vegetans	Labial gingiva, buccal and labial mucosa; less common: tongue, soft and hard palate	Erythematous and thickened oral mucosa with multiple pustules and superficial erosions
Angular cheilitis	Corner of the oral cavity	Erythema at the corners of the mouth with or without painful fissures and sores
Others: Glossitis Periodontitis and dental caries Perioral dermatitis Recurrent buccal abscesses Submandibolar lymphoadenopathy Salivary duct fistula	Oral mucosa, gingiva, tongue, teeth, periodontal tissue, alveolar bone, perioral skin, palate, lips, lymph nodes, salivary glands	
